# Growth hormone in adipose dysfunction and senescence

**DOI:** 10.18632/oncotarget.3997

**Published:** 2015-05-05

**Authors:** Michael B. Stout, Tamara Tchkonia, James L. Kirkland

**Affiliations:** Robert and Arlene Kogod Center on Aging, Mayo Clinic, Rochester, MN, USA

Growth hormone (GH) is an important modulator of maturation, adiposity, and metabolism in mammals. It also has profound effects on age-related disease onset and healthspan [1]. GH-deficient and -resistant mice have up to a 78% increase in maximum lifespan and are protected from age-related metabolic dysfunction and cancer [2]. Although lifespan does not appear to be extended in humans with GH resistance, these individuals are protected from a variety of age-related pathologies, particularly diabetes and cancer [3]. Several mechanisms have been proposed and likely contribute to the pro-survival effects of curtailed GH action, including enhanced stress resistance, xenobiotic metabolism, and insulin sensitivity in combination with reduced inflammation [1]. In contrast, both mice and humans with excess GH develop phenotypes indicative of premature aging and have greater disease burden and mortality risk [1, 2]. Furthermore, mice and humans with altered GH action also display profound changes in adipose tissue homeostasis and lipid distribution. GH-deficient and -resistant mice preferentially deposit lipid in subcutaneous adipose depots, whereas humans with Laron Syndrome exhibit markedly increased subcutaneous and visceral adiposity [1, 2]. Despite the development of obesity, the overwhelming majority of these mice and human subjects are safeguarded from metabolic dysfunction. Conversely, transgenic GH-overexpressing mice and human acromegaly patients remain lean throughout life, yet often develop insulin resistance and an elevated pro-inflammatory status [1]. These observations suggest GH may accelerate declines in adipose tissue homeostasis, which has previously been linked with age-related perturbations in systemic inflammation and metabolism [4].

Adipose tissue functional capacity declines with advancing age [4]. This deterioration is particularly evident in subcutaneous depots, which promotes increased visceral adiposity, ectopic lipid deposition, and concomitant metabolic disturbances. Preadipocyte proliferation and differentiation potential are curtailed with aging and result in reduced lipid deposition in subcutaneous adipose tissue [4]. Pro-inflammatory cytokines, chemokines, and extracellular matrix proteases are believed to play a central role in preadipocyte dysfunction and adipose tissue remodeling with aging. Senescent cells, which possess a highly pro-inflammatory secretome, accumulate in adipose tissue with advancing age, and thus, have emerged as a potential contributor to adipose tissue dysfunction [4]. Interestingly, the elimination of senescent cells preserves adipose tissue mass and enhances healthspan in mice with an accelerated aging phenotype [5]. Given that GHdeficient and -resistant mice appear to be protected from age-related lipid redistribution and metabolic dysfunction, we hypothesized that GH action would be predictive of subcutaneous adipose tissue lipid storage capacity and senescent cell burden. To test our hypothesis, we evaluated adipose tissue functional capacity and senescent cell accumulation in several models with altered GH action.

Our study, now published in *Aging*, establishes a relationship between GH action, lipid deposition, and adipose tissue senescent cell accumulation [6]. To start, we confirmed that both GH-deficient and -resistant models are protected from age-related lipid redistribution as evidenced by greater subcutaneous to visceral adipose tissue ratios than their respective control littermates at 18 months of age. These findings were supported by the observation that GH-resistant mice also accumulated less hepatic triglycerides during this time. We subsequently determined that primary subcutaneous preadipocytes from 20-month old GH-resistant mice possess greater differentiation capacity than those from age-matched controls; providing a potential explanation for enhanced lipid storage capacity of subcutaneous adipose tissue in mice with curtailed GH action. Considering the close connection between inflammatory status, adipose tissue homeostasis, and GH action, we sought to determine if our previous findings were associated with adipose tissue senescent cell accumulation. We found that GH-deficient and -resistant mice exhibited far less senescent cell burden in several adipose tissue depots compared to control littermates at 18 months of age. Conversely, GHoverexpressing (10 months of age) and GH-injected (19 months of age) mice accumulated more senescent cells within adipose tissue than their controls, which we later determined was similar to 24-month old chronologically aged mice.

We conclude that senescent cell accumulation in adipose tissue is linked with GH activity and may play a key role in age-related adipose tissue dysfunction. It is currently unclear if GH directly promotes cellular senescence in adipose tissue through geroconversion [7], or if GH-mediated effects on insulin-like growth factor-1 (IGF-1) and insulin signaling pathways play a more dominant role [1, 2]. Future investigation of the role these distinct, yet related, signaling pathways play in the emergence of cellular senescence will provide insights into links among aging, cellular senescence, and adipose tissue dysfunction. Studies using a new class of drugs that selectively clear senescent cells, senolytic agents [8], in experimental animals and eventually humans with obesity and excess or diminished GH and IGF-1 action will be a path towards testing if these links are causal.
